# Recognition and confirmation of key genes associated with nicotinamide metabolism in acute myocardial infarction

**DOI:** 10.1186/s41065-026-00671-0

**Published:** 2026-03-29

**Authors:** Nan Qu, Fawen Bai, Jin Lan

**Affiliations:** Department of Cardiology, Ruikang Hospital Affiliated to Guangxi University of Chinese Medicine, Nanning, Guangxi Province China

**Keywords:** Acute myocardial infarction, Nicotinamide metabolism, Mendelian randomization, Immune infiltration analysis

## Abstract

**Background:**

The role of nicotinamide in improving cardiac function suggests its therapeutic benefit for acute myocardial infarction (AMI). This study aims to identify key genes associated with nicotinamide metabolism in AMI, offering novel insights into its therapeutic management.

**Methods:**

The GSE60993 and GSE61144 datasets were retrieved from the database. Mendelian randomization (MR) analysis, Cytoscape-plugin cytoHubba algorithms, and expression validation were used to identify key genes linked to nicotinamide metabolism. Subcellular localization, gene set enrichment analysis (GSEA), immune infiltration analysis, drug prediction, and molecular docking were then performed. Finally, the expression of key genes was validated experimentally in clinical samples.

**Results:**

Three key genes (FLT3LG, ITGB7, and PARP1) were found to be causally associated with AMI, ranking in the top three of the three Cytoscape-plugin cytoHubba algorithms. FLT3LG and ITGB7 were identified as risk factors (odds ratio [OR] > 1, P-value < 0.05), while PARP1 was a protective factor (OR < 1, P-value < 0.05). Expression analysis in GSE60993 and GSE61144 confirmed similar trends for these genes. FLT3LG and ITGB7 were predominantly located in the cytoplasm, whereas PARP1 was mainly in the nucleus. GSEA results indicated involvement of these genes in pathways such as the ribosome, oxidative phosphorylation, and spliceosome. Immune infiltration analysis showed that neutrophil scores were higher in AMI and negatively correlated with ITGB7 (cor = -0.84), PARP1 (cor = -0.75), and FLT3LG (cor = -0.86). Drug prediction and molecular docking revealed strong correlations between the key genes and Tetradioxin, with excellent binding ability. Expression trends of the three genes in clinical samples aligned with bioinformatics findings.

**Conclusions:**

This study identified three genes associated with nicotinamide metabolism whose altered expression or function may have played a role in the pathological process of AMI. These genes could provide potential reference points for the diagnosis and treatment of AMI.

**Supplementary Information:**

The online version contains supplementary material available at 10.1186/s41065-026-00671-0.

## Introduction

Acute myocardial infarction (AMI) refers to myocardial necrosis resulting from persistent ischemia and hypoxia due to the acute occlusion of the coronary artery. The pathological hallmark of AMI is the rupture or erosion of coronary atherosclerotic plaques, followed by thrombus formation, which leads to a significant reduction or cessation of coronary blood flow, causing irreversible damage to myocardial cells [1]. It is important to note that reperfusion therapies, such as percutaneous coronary intervention and thrombolytic therapy, have significantly improved patient prognosis. However, long-term complications of AMI, such as myocardial fibrosis, left ventricular remodeling [2], and heart failure [3], place a considerable burden on both individual health and healthcare systems.

Current diagnostic methods for AMI, primarily based on clinical symptoms, electrocardiograms (ECGs), and serum biomarkers, have notable limitations. Some patients present with atypical clinical symptoms, increasing the risk of misdiagnosis or missed diagnosis. Additionally, patients with acute coronary artery occlusion may not exhibit ST-segment elevation [4]. Although high-sensitivity cardiac troponin (hs-cTn) has improved diagnostic sensitivity for early AMI and enhanced prognostic capabilities, it also has several drawbacks: it cannot differentiate the etiologies of myocardial injury, there is controversy regarding threshold selection, it requires dynamic monitoring, and its prognostic value can be complex. Clinical application of hs-cTn demands comprehensive judgment, incorporating clinical manifestations and a cautious approach to avoiding false-positive results [5]. Therefore, exploring novel diagnostic methods and discovering new biomarkers are key steps toward advancing precision medicine in AMI.

Nicotinamide (NAM), an amide form of vitamin B3, plays an essential role in human physiology. It acts as a precursor to nicotinamide adenine dinucleotide (NAD) and NAD phosphate (NADP), both of which are essential for various cellular processes. NAD and NADP are involved in energy metabolism, redox reactions, and DNA repair, all of which are vital for maintaining normal cellular functions [6]. However, disruptions in NAM metabolism can have significant consequences. Abnormalities in key enzymes involved in the NAM metabolic pathway can impair the synthesis of NAD and NADP, thereby compromising cellular energy supply and DNA repair mechanisms, which are essential for cell survival and function [7]. Differential metabolites are predominantly concentrated in nicotinic acid and NAM metabolic pathways in myocardial infarction [8], strongly suggesting that NAM metabolic disorders may contribute to the pathophysiology of AMI by affecting myocardial energy metabolism, oxidative stress responses, and DNA repair.

Mendelian randomization (MR) is a genetic instrumental variable (IV) method that leverages Mendelian inheritance principles to infer causal relationships [9]. By using genetic variants, such as single nucleotide polymorphisms (SNPs), strongly associated with exposures as IVs, MR mimics the randomization of clinical trials, addressing confounding and reverse causation in observational studies [10]. Key advantages of MR include: (1) Random allocation of genetic variants during meiosis, approximating natural randomization; (2) Cost-effective analysis using existing Genome-wide association study (GWAS) data; and (3) The ability to assess lifelong exposure effects. MR identifies disease-causal genes by analyzing genetic loci associated with phenotypes [10], such as PCSK9 in LDL cholesterol regulation and cardiovascular risk [11]. Integration with multi-omics data further enhances causal inference accuracy, providing robust insights into disease mechanisms and aiding in therapeutic target discovery. However, no studies have yet utilized MR analysis to investigate key genes associated with NAM metabolism in AMI.

Thus, this study aims to identify key NAM-related genes in AMI through differential expression analysis, MR analysis, and expression validation. Additionally, the underlying mechanisms of these genes will be explored using Gene Set Enrichment Analysis (GSEA), molecular regulatory network construction, and drug prediction approaches.

## Materials and methods

### Data collection

The datasets related to AMI were retrieved from the Gene Expression Omnibus (GEO) database (http://www.ncbi.nlm.nih.gov/geo/). The dataset GSE60993 (platform GPL6884) contained blood specimens from 17 patients with AMI and 7 healthy controls, with 9 unstable angina samples excluded, and was designated as the training set. The validation set GSE61144 (platform GPL6106) included blood samples from 7 patients with AMI and 10 healthy controls, with STEMI samples from 7 recovered cases removed. The inclusion criteria for AMI patients were those undergoing primary percutaneous coronary intervention for acute coronary syndrome, with samples collected within 4 h of chest pain onset. The healthy control group was explicitly defined as subjects confirmed via coronary angiography to have no coronary artery abnormalities [12] Additionally, 42 NAM metabolism-related genes (NMRGs) were integrated from a reference [13] (Additional file 1). GWAS data for AMI (ukb-a-533) were retrieved from the Integrative Epidemiology Unit Open GWAS (IEU OpenGWAS) database (https://gwas.mrcieu.ac.uk/), including 337,199 European samples (3,927 cases and 333,272 controls) and 10,894,596 SNPs. Expression quantitative trait loci (eQTLs) for exposure factors were also obtained from the IEU OpenGWAS database.

### Differential expression analysis

To identify differentially expressed genes (DEGs) between disease and control samples within the training set, the ‘limma’ package (v 3.19) [14] was used for differential expression analysis, with DEGs screened based on P-value < 0.05 and |log_2_ fold change (FC)| > 0.5. The ‘ggplot2’ package (v 3.4.4) [15] was used to generate a volcano plot to visualize the DEGs, while the ‘ComplexHeatmap’ package (v 2.18.0) [16] was used to create a heatmap.

### Weighted gene co-expression network analysis (WGCNA) and identification of candidate genes

To compare the differences in NMRG scores between disease and control samples, the single-sample Gene Set Enrichment Analysis (ssGSEA) algorithm in the ‘GSVA’ package (v 1.46.0) [17] was employed to calculate NMRG scores for each sample based on the training set. The differences in scores between disease and control groups were analyzed using the Wilcoxon test (P-value < 0.05).

To identify module genes involved in NAM metabolism, WGCNA was performed on the NMRG scores using the ‘WGCNA’ package (v 1.71) [18] for all samples in the training set. Samples were initially clustered by hierarchical clustering using Euclidean distances of expression levels, and outliers were identified and excluded. To ensure intergenic interactions maximized a scale-free distribution, the soft threshold (β) was calibrated to achieve an R^2^ > 0.8 and mean connectivity near zero. A scale-free network was established based on the calibrated soft threshold, and genes were divided into several modules, each assigned a different color (minimum of 300 genes per module, module merge threshold 0.25). The correlation between NMRG scores and the modules was analyzed to identify one module with the highest positive correlation and one with the highest negative correlation with NMRG scores as the key modules (|correlation coefficient (cor)| ≥ 0.3, P-value < 0.05). Finally, to identify key module genes, within-module analysis was performed, selecting genes with |module membership (MM)| > 0.8 and |gene significance (GS)| > 0.8.

To identify the DEGs related to NAM metabolism in AMI, the ‘ggVenndiagram’ package (v 0.1.10) [19] was used to intersect DEGs and key module genes. The intersecting genes were considered candidate genes for further analysis.

### Mendelian randomization (MR) analysis

To investigate the potential causal relationship between each candidate gene and AMI, MR analysis was performed, setting candidate genes as exposures and AMI as the outcome. To ensure reliable results, the following three assumptions were met: 1) Genetic variation in IVs must be strongly correlated with exposure factors; 2) Genetic variation in IVs should not be associated with confounding factors; and 3) Genetic variation in IVs should influence the outcome only through the exposure factors. To select IVs significantly associated with exposure factors, the extract_instruments function in ‘TwoSampleMR’ (v 0.5.6) [20] was used to read exposure factors and select IVs (P-value < 5 × 10^–8^, clump = TRUE, r^2^ = 0.001, kb = 100). SNPs with significant associations with AMI and F-statistic values < 10 were removed, and exposure factors with fewer than 3 SNPs were excluded. The harmonise_data function in ‘TwoSampleMR’ (v 0.5.6) was then used to harmonize effect alleles and effect sizes. Data on exposure factors and outcomes were combined using this function, and the merged data were used for MR analysis.

Various MR approaches, including MR Egger [21], weighted median [22], inverse variance weighted (IVW) [23], weighted mode [24], and simple mode [25], were employed to validate the causal relationships between exposure factors and the outcome, with the IVW method providing the primary results (P-value < 0.05). Exposure factors with an odds ratio (OR) greater than 1 were considered risk factors, while those with an OR less than 1 were deemed protective factors. To further illustrate the MR results, scatter plots, forest plots, and funnel plots were generated. A heterogeneity test was performed using the mr_heterogeneity function in ‘TwoSampleMR’ (v 0.5.6) [20], and Cochran’s Q-statistic was computed *via* the IVW method. A P-value > 0.05 indicated no heterogeneity. When significant heterogeneity was detected (P < 0.05), we employed a random-effects IVW model as the primary analysis method to obtain a more conservative effect estimate. Horizontal pleiotropy was assessed using the mr_pleiotropy_test function in ‘TwoSampleMR’ (v 0.5.6) [20] and the mr_presso function in ‘MRPRESSO’ (v 0.6.4) [26], with a P-value > 0.05 indicating no horizontal pleiotropy. A Leave-One-Out (LOO) test was conducted using the mr_leaveoneout function to assess the robustness and reliability of the MR results. The Steiger test was also performed (P-value < 0.05) to verify the directionality of the MR results. Genes passing directionality testing were selected as feature genes.

### Functional analysis of feature genes and construction of protein-protein interaction (PPI) network

Gene Ontology (GO) and Kyoto Encyclopedia of Genes and Genomes (KEGG) enrichment analyses were conducted using the ‘clusterProfiler’ package (v 4.10.0) [27], with P-value < 0.05 to identify the most significantly enriched items. To investigate gene interactions at the protein level, feature genes were input into the Search Tool for the Retrieval of Interacting Genes (STRING) database (https://string-db.org/) with a confidence score > 0.15. The resulting PPI network was visualized using ‘Cytoscape’ software (v 3.10.2) [28].

### Screening of candidate key genes

The three algorithms—density of maximum neighborhood component (DMNC), maximum clique centrality (MCC), and edge percolated component (EPC)—from the Cytoscape-plugin cytoHubba were employed to identify the top three genes based on the scores of each algorithm. The ‘ggVenndiagram’ package (v 0.1.10) [28] was then used to intersect the top three genes from each algorithm, and the intersecting genes were selected as candidate key genes.

### Identification and correlation analysis of key genes

To assess whether the expression patterns of the candidate key genes were consistent across both the validation and training sets, the Wilcoxon test was applied (P-value < 0.05). Genes with differing expression levels between disease and control samples, but consistent trends in both datasets, were identified as key genes.

Further, Spearman correlation analysis was performed to examine the relationships between key genes (|cor| > 0.3, P-value < 0.05) using the ‘psych’ package (v 2.4.1) [29].

### Chromosome and subcellular localization

To map the chromosomal positions of the key genes, the ‘RCircos’ package (v 1.2.2) [30] was used, along with human chromosome data from the UCSC Genome Browser (https://hgdownload.cse.ucsc.edu/goldenpath/hg19/bigZips/). For predicting the subcellular localization of the key genes, the mRNALocater database (http://bio-bigdata.cn/mRNALocater/) was employed. Gene sequences retrieved from the National Center for Biotechnology Information (NCBI) (https://www.ncbi.nlm.nih.gov/) were input into the mRNALocater database for this prediction.

### Establishment and evaluation of a nomogram

A diagnostic nomogram was constructed to predict the prevalence rate of AMI in the training set using the ‘rms’ package (v 6.9.0) [31] based on the key genes. To evaluate the prediction performance of the nomogram, decision curve analysis (DCA) and calibration curves were plotted using the ‘rmda’ package (v 1.6) [31] and ‘rms’ package (v 6.9.0) [32].

### Gene set enrichment analysis (GSEA) and gene set variation analysis (GSVA)

To explore the functional pathways associated with key genes in AMI, GSEA was performed on the key genes. The reference gene set ‘c2.cp.kegg.v2023.1.Hs.symbols.gmt’ was selected from the Molecular Signatures Database (MSigDB, https://www.gsea-msigdb.org/gsea/msigdb). Initially, Spearman correlation analyses were conducted between each key gene and all other genes in the training set using the ‘psych’ package (v 2.4.1) [29] to calculate the correlation coefficients. Genes were then ranked in descending order based on these coefficients. The sorted data were used to perform GSEA (|normalized enrichment score (NES)| > 1, P-value < 0.05) using the ‘clusterProfiler’ package (v 4.10.0) [27]. Significant pathways were visualized using the ‘enrichplot’ package (v 1.18.4) [33].

Additionally, GSVA was conducted in the training set using the ‘GSVA’ package (v 1.46.0) [17]. The ‘c2.cp.kegg.v7.2.symbols.gmt’ gene set was used as the reference set from MSigDB. Differential analysis was performed between disease and control samples using the ‘limma’ package (v 3.19) [14]. Enrichment scores for signaling pathways were evaluated, and significant P-values (P-value < 0.05) were calculated to highlight the top 10 pathways with positive and negative scores.

### Immune infiltration analysis

To investigate immune infiltration in different samples in the training set, the ssGSEA scores for 28 immune cell types [34] were calculated using the ssGSEA algorithm in the ‘GSVA’ package (v 1.46.0) [17]. The Wilcoxon test was applied to compare the ssGSEA scores between disease and control samples (P-value < 0.05), with differential immune cells identified and visualized using the ‘ggplot2’ package (v 3.4.4) [15]. Additionally, Spearman correlation analysis was performed to assess the relationships between differential immune cells and key genes (|cor| > 0.3, P-value < 0.05) using the ‘psych’ package (v 2.4.1) [29].

### Construction of molecular regulatory network

To explore the regulatory complexity of key gene expression, molecular regulatory networks were constructed. The get_multimir function of the ‘multiMiR’ package (v 3.19) [35] was used to predict miRNAs and the lncRNAs upstream of these miRNAs. The miRNA-mRNA and lncRNA-miRNA-mRNA regulatory network was then generated and visualized using ‘Cytoscape’ software (v 3.10.2) [28].

### Drug prediction and molecular docking

To investigate potential drugs for treating AMI and explore the associations between key genes and drugs, the Drug Signatures Database (DSigDB) (https://www.dsigdb.org/) was utilized to predict drugs potentially linked with key genes (P-value < 0.05). The top 10 drug-key gene networks were visualized using ‘Cytoscape’ software (v 3.10.2) [28].

To further validate the binding affinity between drugs and key genes, molecular docking was performed between each key gene and its corresponding drug active ingredient. Drugs with the highest correlation score to the key genes were selected. The 3D structure of the drug active ingredient was first retrieved from the PubChem database (https://pubchem.ncbi.nlm.nih.gov/). Next, the 3D protein structure of the key gene-associated protein was obtained from the RCSB Protein Data Bank (RCSB PDB) (https://www.rcsb.org/). Potential docking sites within the target protein were predicted using CB-Dock2 (https://cadd.labshare.cn/cb-dock2/php/index.php), followed by docking simulations and visualization of the results.

### Reverse Transcription-quantitative Polymerase Chain Reaction (RT-qPCR)

Five AMI blood specimens and 5 control blood specimens were collected from the Ruikang Hospital Affiliated to Guangxi University of Chinese Medicine. Ethical approval for the study was granted by the hospital’s ethics committee (approval number: KY2025-143). RNA concentration was measured using the NanoPhotometer N50, and cDNA synthesis was performed using the Hifair^®^Ⅲ 1st Strand cDNA Synthesis SuperMix. Primers were synthesized by a biological company (Table 1). RT-qPCR was conducted with GAPDH as the internal reference gene, and the expression levels of the key genes were calculated using the 2^−ΔΔCt^ method. ‘GraphPad Prism’ (v 5.0.0) [36] was used to plot the data and calculate the P-values. Differences between the experimental groups were assessed using the t-test (P-value < 0.05).

**Table 1:** Primer sequences

Primer	Sequences
FLT3LG F	TCACTTTCGGTCTCTGGCTG
FLT3LG R	CAGACTGCGGGAGAAAACAG
ITGB7 F	GCACAGAGTTTGAGCTGAGTAA
ITGB7 R	TCTGGTTGATTCGGACGTGG
PARP1 F	TGTCCTGGAGAAAGGTGGGA
PARP1 R	TCCTTGGACGGCATCTGTTC
GAPDH F	ATGGGCAGCCGTTAGGAAAG
GAPDH R	AGGAAAAGCATCACCCGGAG

### Statistical analysis

Bioinformatics analyses were performed using R programming language (v 4.2.2). The differences between the two groups were evaluated using the Wilcoxon test, with a significance threshold of P-value < 0.05 considered statistically significant.

## Results

### There were 131 candidate genes associated with nicotinamide metabolism

**Fig. 1:**
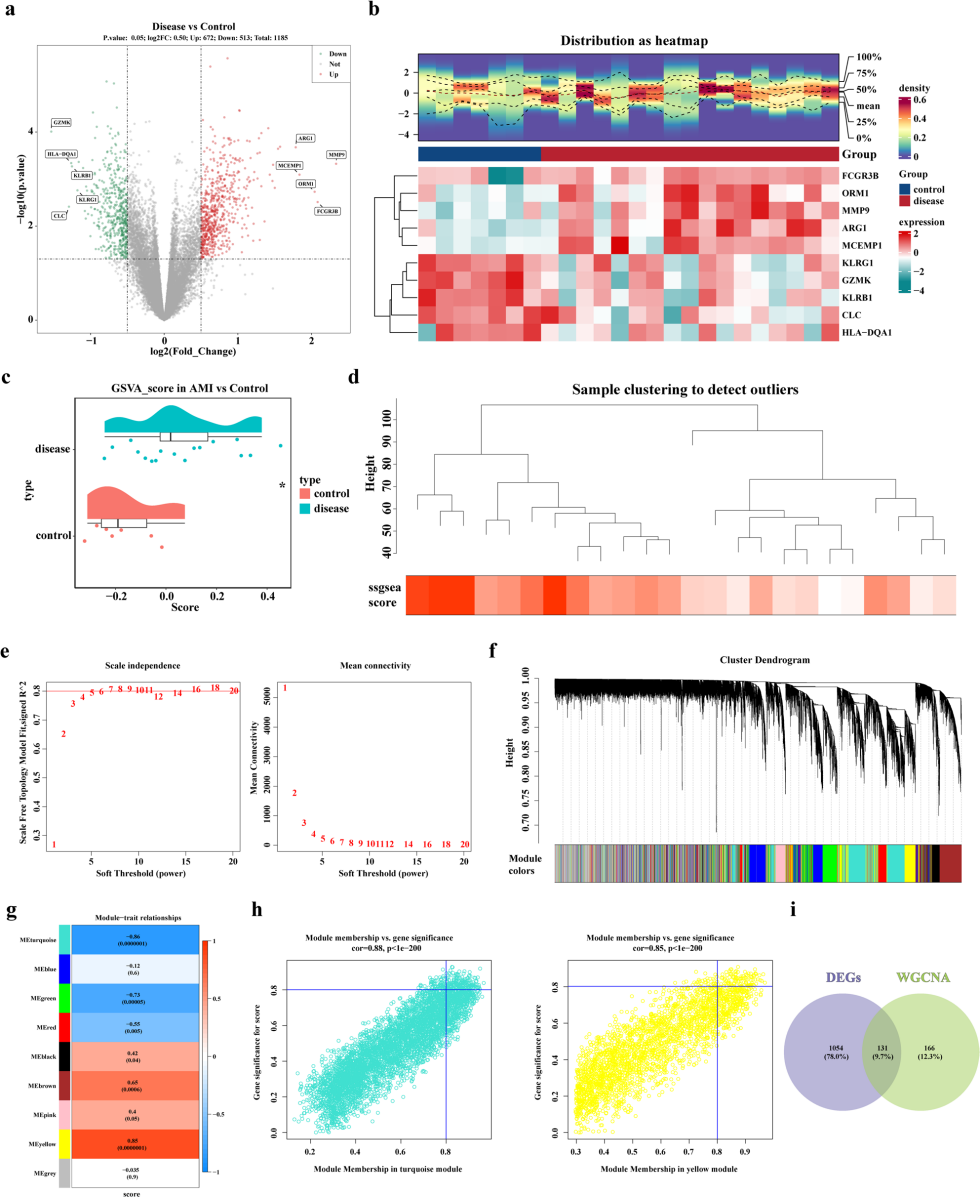
Identification of nicotinamide metabolism-related genes in AMI through differential expression analysis and WGCNA. **a** Volcano plot of DEGs. The y-axis represents -log10 (p-value), and the x-axis shows the log2 fold change. Green dots indicate downregulated genes, red dots represent upregulated genes, and gray dots denote non-significant genes. **b** Heatmap of DEGs. The upper section displays expression density heatmaps of the top 10 genes with the largest fold changes, showing five quantiles and mean expression lines. The lower section presents the expression heatmap, where each column represents a sample and each row shows gene expression levels across samples. **c** ssGSEA scores for NMRGs. Asterisks indicate statistical significance (* *p* < 0.05, ** *p* < 0.01, *** *p* < 0.001). **d** Hierarchical clustering of samples. The dendrogram branches represent individual samples, with the red line indicating the cut-off threshold. The y-axis shows the Euclidean distance of sample expression profiles. **e** Soft threshold selection for WGCNA. The left plot displays the scale-free fit index (red line indicates the chosen threshold). The right plot illustrates network connectivity under different soft thresholds. **f** Dynamic tree cut for module identification. The upper panel shows the gene hierarchical clustering dendrogram, while the lower panel depicts assigned gene modules. Closely clustered genes are grouped into the same module. **g** Module-trait correlation heatmap. The left color bar represents modules, and the right color scale indicates correlation strength. Darker shades in the heatmap denote higher correlations, with red for positive and blue for negative associations. **h** Scatter plot of GS versus MM for key modules. GS reflects the correlation between genes and the trait, while MM represents gene-module connectivity

A total of 1,185 DEGs were identified between the AMI and control samples in the training set, including 672 up-regulated genes and 513 down-regulated genes (Fig. 1a-b). To identify key module genes associated with NAM metabolism in AMI, ssGSEA scores for NMRGs showed significant differences between AMI and control samples (P-value < 0.05) (Fig. 1c). Based on this, when β was set to 7, a total of 9 modules were obtained (Fig. 1d-f). The Module Eigengene (ME) yellow and ME turquoise modules, which strongly correlated with NMRGs (cor = 0.85, P-value < 0.001 and cor = -0.86, P-value < 0.001), were selected by WGCNA, and 297 key module genes were identified (Fig. 1g-h, Additional file 2). To further identify DEGs associated with NAM metabolism, 1,185 DEGs and 297 key module genes were intersected, resulting in 131 intersection genes, which were selected as candidate genes (Fig. 1i).

### A total of 17 feature genes causally related to AMI

To examine the causal relationships between exposure factors and AMI, the IVW algorithm was applied based on eligible exposure factors. A total of 17 exposure factors with a significant causal relationship with AMI were identified, of which 5 were protective factors (OR < 1) and 12 were risk factors (OR > 1) (Table 2). The scatter plot revealed a negative correlation between 5 exposure factors and AMI (slope < 0), while the remaining exposure factors showed a positive correlation with AMI (slope > 0). Notably, the intercepts were near 0, indicating that these associations were not significantly influenced by confounding factors (Additional file 3a-q). The forest plot confirmed that the effect size of the 5 protective factors was less than 0, while the effect size of the 12 risk factors was greater than 0, confirming that 5 exposure factors acted as protective factors and 12 as risk factors for AMI (Additional file 4a-q). The funnel plot indicated that the 17 exposure factors adhered to Mendel’s second law of random grouping (Additional file 5a-q).

**Table 2:** Causation between exposure factors and outcome

id.exposure	Gene	id.outcome	nsnp	pval	OR
eqtl-a-ENSG00000090554	FLT3LG	ukb-a-533	6	<0.001	1.014
eqtl-a-ENSG00000104490	NCALD		17	0.008	0.998
eqtl-a-ENSG00000105352	CEACAM4		12	0.007	0.998
eqtl-a-ENSG00000107679	PLEKHA1		10	0.041	1.001
eqtl-a-ENSG00000110651	CD81		4	0.018	1.002
eqtl-a-ENSG00000125166	GOT2		8	<0.001	1.002
eqtl-a-ENSG00000134686	PHC2		6	0.022	0.998
eqtl-a-ENSG00000135766	EGLN1		6	0.00028	1.002
eqtl-a-ENSG00000138378	STAT4		3	0.001	1.014
eqtl-a-ENSG00000139626	ITGB7		16	0.038	1.001
eqtl-a-ENSG00000140526	ABHD2		8	0.030	1.002
eqtl-a-ENSG00000143799	PARP1		21	0.035	0.999
eqtl-a-ENSG00000145491	ROPN1L		6	0.001	1.004
eqtl-a-ENSG00000173511	VEGFB		4	0.005	1.003
eqtl-a-ENSG00000186918	ZNF395		3	0.004	1.005
eqtl-a-ENSG00000188315	C3orf62		14	0.018	0.998
eqtl-a-ENSG00000197208	SLC22A4		19	0.022	1.000

A sensitivity analysis was performed to assess the reliability of the MR results. The heterogeneity test revealed that most exposures had P-values exceeding 0.05, suggesting no heterogeneity between these exposures and the outcome. However, significant heterogeneity was detected for FLT3LG (*P* = 0.006) and STAT4 (*P* = 0.007) (Table 3). Consequently, a random-effects IVW model was employed for primary causal effect estimation in these two genes, providing more conservative estimates by incorporating heterogeneity. The P-values for horizontal pleiotropy were above 0.05, indicating no confounding factor interference (Table 4). The LOO test showed no significant changes in the effect size of IVs (Additional file 6a-q). Finally, the Steiger test for all 17 exposures yielded a result of TRUE (Table 5), confirming the directionality of the results. These 17 genes were identified as feature genes.

**Table 3:** Heterogeneity test

Gene	Method	Q	Q_df	*P*-value
NCALD	Inverse variance weighted	15.725	16.000	0.472
CEACAM4		14.585	11.000	0.202
PLEKHA1		8.495	9.000	0.485
CD81		3.098	3.000	0.377
GOT2		3.569	7.000	0.828
EGLN1		2.533	5.000	0.772
STAT4		9.820	2.000	0.007
ITGB7		10.365	15.000	0.796
ABHD2		5.290	7.000	0.625
ROPN1L		5.299	5.000	0.380
VEGFB		1.808	3.000	0.613
ZNF395		0.493	2.000	0.782
C3orf62		9.099	13.000	0.765
SLC22A4		11.553	18.000	0.869
FLT3LG		16.000	5.000	0.006
PHC2		5.914	5.000	0.315
PARP1		8.694	20.000	0.986

**Table 4:** Horizontal pleiotropic activity test

Gene	egger_intercept	se	*P*-value
FLT3LG	0.00196	0.00092	0.099
NCALD	0.00003	0.00013	0.824
CEACAM4	-0.00008	0.00023	0.739
PLEKHA1	0.00030	0.00026	0.287
CD81	0.00007	0.00044	0.887
GOT2	0.00002	0.00041	0.970
PHC2	-0.00068	0.00035	0.129
EGLN1	0.00050	0.00038	0.259
STAT4	-0.00262	0.00406	0.635
ITGB7	0.00006	0.00021	0.777
ABHD2	0.00048	0.00029	0.153
PARP1	-0.00024	0.00022	0.286
ROPN1L	-0.00064	0.00075	0.439
VEGFB	-0.00068	0.00064	0.400
ZNF395	-0.00088	0.00131	0.624
C3orf62	-0.00021	0.00065	0.753
SLC22A4	-0.00018	0.00017	0.292

**Table 5:** Steiger reverse causality detection

id.exposure	id.outcome	snp_r2.exposure	snp_r2.outcome	correct_causal_direction	*P*-value	SYMBOL
eqtl-a-ENSG00000104490	ukb-a-533	0.071108	0.000067	TRUE	0	NCALD
eqtl-a-ENSG00000105352		0.161754	0.000071	TRUE	0	CEACAM4
eqtl-a-ENSG00000107679		0.071765	0.000038	TRUE	0	PLEKHA1
eqtl-a-ENSG00000110651		0.035608	0.000026	TRUE	1.10E-183	CD81
eqtl-a-ENSG00000125166		0.066851	0.000068	TRUE	0	GOT2
eqtl-a-ENSG00000135766		0.049120	0.000047	TRUE	3.54E-234	EGLN1
eqtl-a-ENSG00000138378		0.007890	0.000287	TRUE	6.25E-28	STAT4
eqtl-a-ENSG00000139626		0.104403	0.000043	TRUE	0	ITGB7
eqtl-a-ENSG00000140526		0.038086	0.000030	TRUE	2.02E-179	ABHD2
eqtl-a-ENSG00000145491		0.012140	0.000051	TRUE	2.62E-57	ROPN1L
eqtl-a-ENSG00000173511		0.012753	0.000028	TRUE	3.71E-75	VEGFB
eqtl-a-ENSG00000186918		0.005645	0.000026	TRUE	2.82E-29	ZNF395
eqtl-a-ENSG00000188315		0.056195	0.000043	TRUE	3.20E-277	C3orf62
eqtl-a-ENSG00000197208		0.314649	0.000050	TRUE	0	SLC22A4
eqtl-a-ENSG00000090554		0.009390	0.000415	TRUE	1.09E-36	FLT3LG
eqtl-a-ENSG00000116353		0.086285	0.000039	TRUE	0	PARP1
eqtl-a-ENSG00000134686		0.032993	0.000036	TRUE	7.71E-191	PHC2

### Key genes were engaged in the regulation of AMI *via* multiple functional pathways

**Fig. 2:**
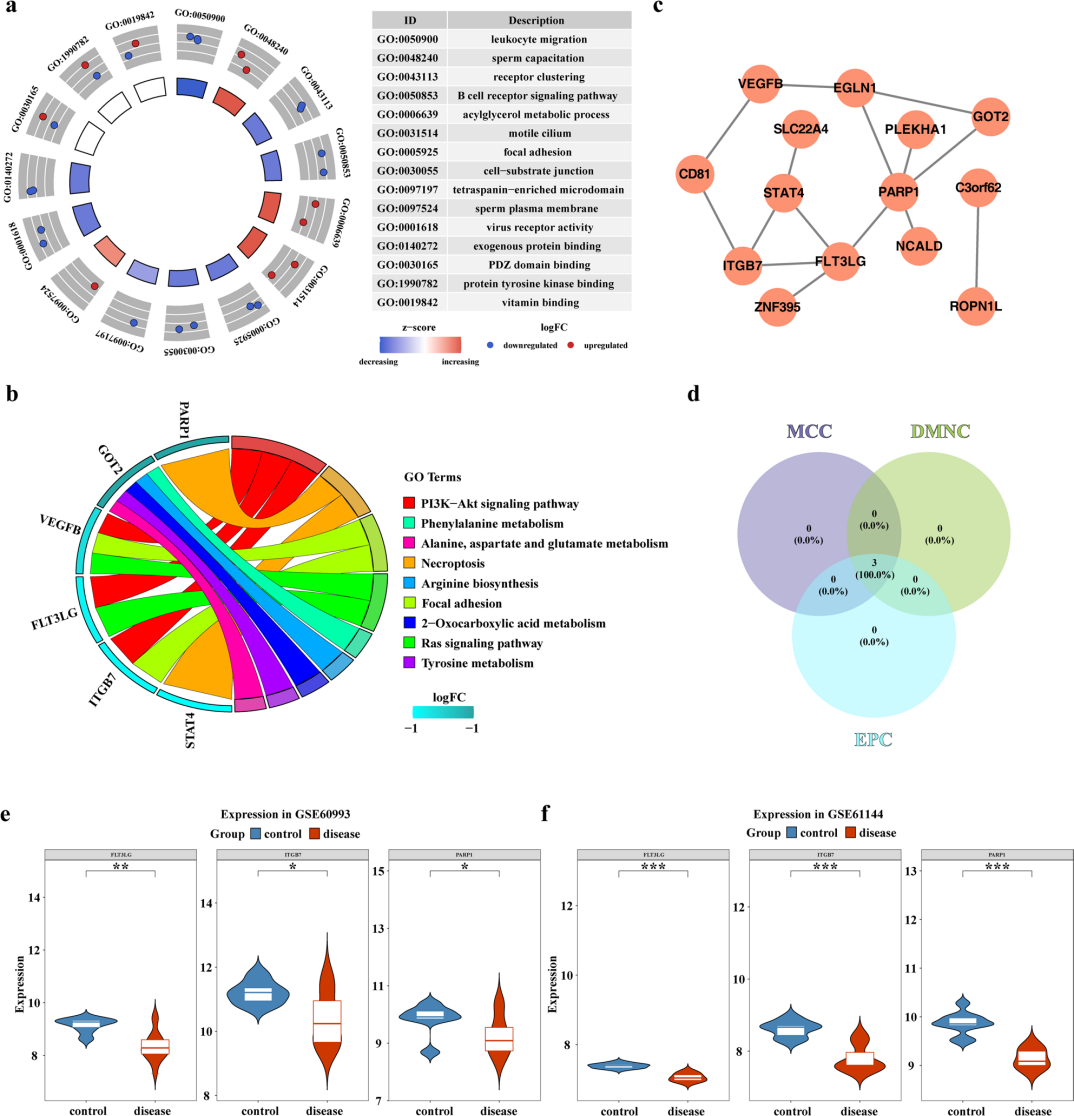
Identification and functional characterization of nicotinamide metabolism-related genes in acute myocardial infarction. **a** GO enrichment analysis of MRGs. The left panel shows a circular plot of GO enrichment analysis, with the inner bar graph indicating the significance of functions (higher bars represent greater significance, and darker colors indicate larger z-scores, suggesting upregulation or downregulation). The outer circle displays scatter plots of gene expression levels in each functional pathway. The right panel provides detailed descriptions of the enriched GO functions. **b** KEGG enrichment pathways of MRGs. The left panel lists enriched gene names, with darker colors representing higher logFC values. The right panel displays the enriched functional pathways, with different colors indicating distinct pathways. **c** PPI protein-protein interaction network. Orange circles represent candidate key genes, and gray connecting lines indicate interactions between proteins. **d** Identification of candidate key genes using cytoHubba. **e** Expression validation of candidate key genes in the training set. Asterisks denote p-values (* *p* < 0.05, ** *p* < 0.01, *** *p* < 0.001). **f** Expression validation of candidate key genes in the validation set

GO enrichment analysis was performed on the feature genes, resulting in 269 pathways, of which 15 were selected for presentation. These included leukocyte migration, B cell receptor signaling pathway, acylglycerol metabolic process, and exogenous protein binding, among others (Fig. 2a, Additional file 7). Additionally, KEGG enrichment analysis identified 9 significant pathways, including tyrosine metabolism, Ras signaling pathway, phenylalanine metabolism, and the PI3K-Akt signaling pathway (Fig. 2b, Additional file 8). In the PPI network, 14 interacting proteins were identified. Among these, PARP1 interacted most frequently with other proteins, such as FLT3LG and EGLN1 (Fig. 2c). Subsequently, three candidate key genes—FLT3LG, ITGB7, and PARP1—were selected based on the DMNC, MCC, and EPC algorithms (Fig. 2d). These genes exhibited significant downregulation in expression levels in the AMI samples within the training set (P-value < 0.05) (Fig. 2e). Furthermore, the expression trend in the validation set was consistent with the training set results (Fig. 2f). Thus, these three genes were considered key genes for subsequent analysis.

### Key genes located on the autosomes and widely distributed in the cytoplasm and nucleus had an excellent diagnostic capability for AMI

**Fig. 3:**
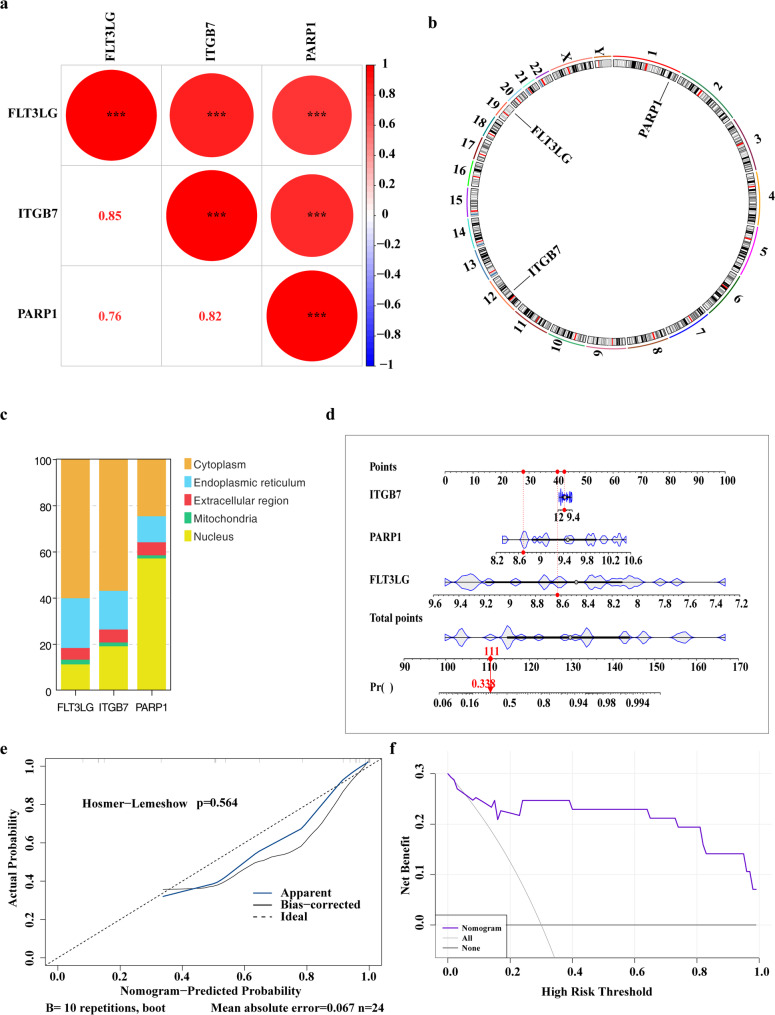
Functional characterization and clinical predictive value of nicotinamide metabolism-related key genes in AMI. **a** Correlation analysis of key genes. The color bar on the right indicates correlation coefficients, with red representing positive correlation and blue representing negative correlation. Numerical values show specific correlation coefficients. Asterisks denote p-values (*p < 0.05, **p < 0.01, ***p < 0.001). **b** Chromosomal localization of key genes. The outer ring displays human chromosomes, while the inner ring marks the positions of key genes. **c** Subcellular localization of key genes. The x-axis represents key genes, and the y-axis shows the percentage distribution across five subcellular compartments. Different colors indicate distinct subcellular locations. **d** Nomogram for key genes. Points represent individual scores for each gene at different expression levels. Total Points indicate the summed score from all genes. **e** Calibration curve for the nomogram model. The x-axis shows the nomogram-predicted probability, and the y-axis represents the actual probability. **f** DCA for the predictive model. The x-axis indicates threshold probability (Pt), where patients are classified as positive and treated if their predicted risk exceeds Pt. The y-axis shows net benefit (NB), calculated as the benefit of intervention minus its harm. The black horizontal line (None) assumes no intervention, while the gray diagonal line (All) assumes all patients receive intervention

Correlation analysis revealed a strong positive relationship between FLT3LG and ITGB7 (cor = 0.85), FLT3LG and PARP1 (cor = 0.76), and ITGB7 and PARP1 (cor = 0.82) (Fig. 3a). Chromosomal localization analysis showed that all three key genes are located on autosomes: PARP1 on chromosome 1, ITGB7 on chromosome 12, and FLT3LG on chromosome 19 (Fig. 3b). Subcellular localization analysis revealed that FLT3LG and ITGB7 were predominantly located in the cytoplasm, with additional distributions in the nucleus, endoplasmic reticulum, extracellular region, and mitochondria. In contrast, PARP1 was primarily localized in the nucleus (Fig. 3c).

A nomogram model was developed using the three key genes to assess their potential in predicting AMI occurrence clinically (Fig. 3d). The calibration curve showed that the model closely followed the line with a slope of 1, indicating good agreement with observed data. In the DCA curve, the nomogram’s prediction model was positioned above the two reference lines (None, All) (Fig. 3e-f), suggesting that the model performs well in predicting AMI incidence.

### Key genes were involved in the regulation of AMI through metabolic pathways and their relevance to immune cells

**Fig. 4:**
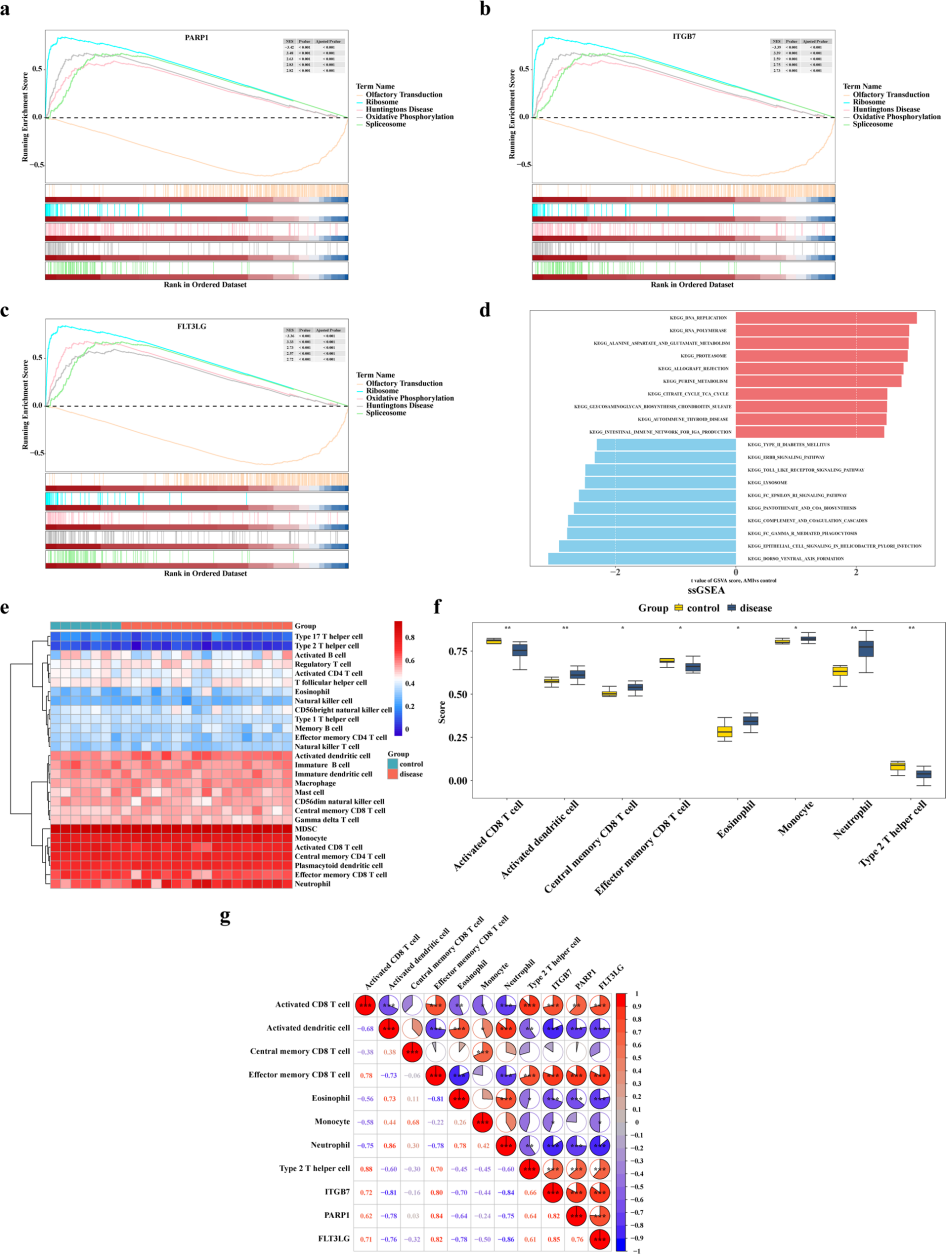
Functional enrichment, immune infiltration, and correlation analysis of nicotinamide metabolism-related key genes in AMI. **a**-**c** GSEA enrichment analysis of key genes. The upper part displays the gene Enrichment Score line plot, where the x-axis represents each gene and the y-axis shows the corresponding Running ES. The peak of the line plot indicates the Enrichment score of the gene set, with genes before the peak representing core genes. The lower part presents hit information, with barcode-like lines marking the positions of genes from the gene set within the background genes, arranged from left to right based on expression levels. **d** GSVA analysis of key genes. Red indicates upregulated gene sets, while blue represents downregulated gene sets. The t-value reflects the enrichment level of each sample in each gene set, with higher GSVA scores indicating more significant expression differences. **e** Abundance of immune cells. Different colors denote distinct cell types, with the y-axis showing the relative proportion of immune cells and each column representing an individual sample. **f** Differential immune cells between AMI and control samples. The x-axis shows immune cells with significant differences, and the y-axis displays their scores. Group differences are highlighted by color, where asterisks denote statistical significance (*p < 0.05, **p < 0.01, ***p < 0.001). **g** Correlation between key genes and differentially infiltrated immune cells. The color bar on the right indicates correlation strength, with red representing positive and blue negative associations. Numerical values specify the correlation coefficients

The GSEA results for the three key genes indicated that FLT3LG was enriched in 119 pathways, ITGB7 in 119 pathways, and PARP1 in 125 pathways (Additional files 9–11). The top 5 enriched pathways for these genes included olfactory conduction, ribosome, oxidative phosphorylation (OXPHOS), Huntington’s disease, and spliceosome pathways (Fig. 4a-c). In addition, GSVA analysis revealed 186 enriched results, with most pathways related to metabolism, such as alanine, aspartate, and glutamate metabolism, purine metabolism, allograft rejection, and glycosaminoglycan biosynthesis chondroitin sulfate (Fig. 4d, Additional file 12).

The ssGSEA score heatmap for 28 immune cell types is shown in Fig. 4e. Eight differential immune cells were identified between the AMI and control groups, including type 2 T helper cells, activated dendritic cells (DCs), neutrophils, effector memory CD8 T cells, eosinophils, monocytes, central memory CD8 T cells, and activated CD8 T cells. The results revealed that AMI had higher neutrophil scores (P-value < 0.01) and lower type 2 T helper cell scores (P-value < 0.01) compared to the control group (Fig. 4f). Spearman correlation analysis demonstrated a strong correlation between the three key genes and the differential immune cells. Notably, ITGB7 showed the most significant positive correlation with activated CD8 T cells (cor = 0.72), effector memory CD8 T cells (cor = 0.80), and type 2 T helper cells (cor = 0.66), while PARP1 exhibited the most pronounced negative correlation with activated DCs (cor = -0.78), eosinophils (cor = -0.64), and neutrophils (cor = -0.75) (P-value < 0.001) (Fig. 4g).

### Multiple molecules and drugs might be related to the key genes in AMI

**Fig. 5:**
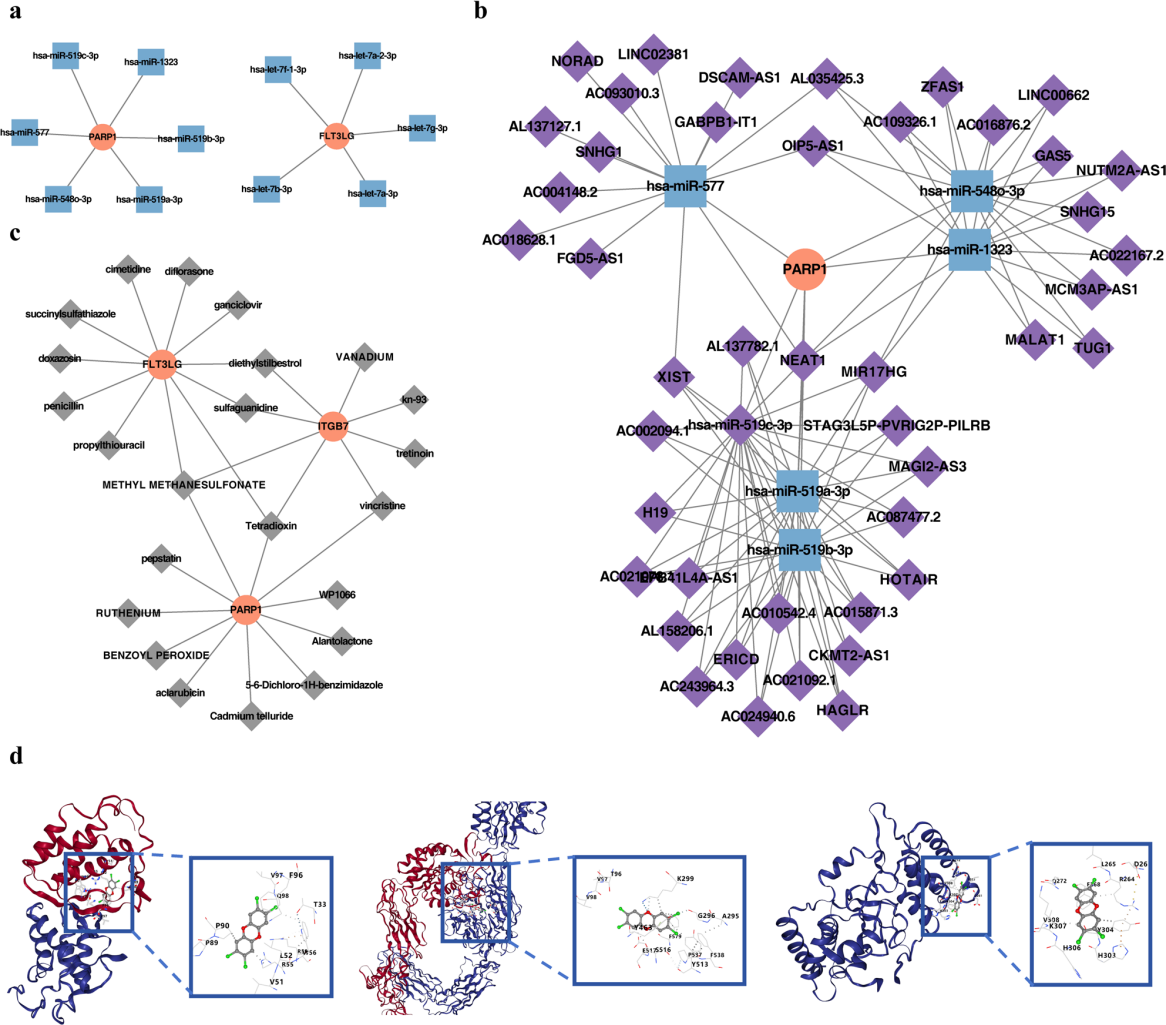
Regulatory networks and molecular docking analysis of nicotinamide metabolism-related key genes in AMI. **a** Regulatory network of miRNA-mRNA interactions. The orange circles represent key genes, while the blue squares indicate miRNAs associated with the expression of these key genes. **b** Regulatory network of lncRNA-miRNA-mRNA interactions. The orange circles denote key genes, the blue squares represent miRNAs linked to key gene expression, and the purple diamonds indicate predicted upstream lncRNAs of these miRNAs. **c** Drug-key gene interaction network. The orange circles represent key genes, and the gray diamonds depict predicted target drugs. **d** Molecular docking models. The left panel shows the three-dimensional structure of the protein corresponding to the key target gene FLT3LG. The middle panel displays the structure for ITGB7, and the right panel illustrates PARP1. The blue boxes highlight the predicted binding sites. The right side of each panel presents the three-dimensional structure of the active drug component Tetradioxin

The results identified 6 miRNAs regulating PARP1 expression, including hsa-miR-519a-3p, hsa-miR-519b-3p, and hsa-miR-1323, and 5 miRNAs regulating FLT3LG expression, such as hsa-let-7a-3p, hsa-let-7 g-3p, and hsa-let-7a-2-3p (Fig. 5a). In addition, 45 lncRNAs were found upstream of miRNAs with a regulatory relationship to PARP1 (Fig. 5b). Based on DSigDB, potential drugs for the key genes were predicted, and the 10 drugs with the strongest correlation to the key genes were selected for display (Fig. 5c). The drug Tetradioxin, which showed the highest correlation score with the three key genes, was chosen for molecular docking. The docking results demonstrated that the binding energies of FLT3LG, ITGB7, and PARP1 with Tetradioxin were − 6.3, -7.0, and − 6.3 kcal/mol, respectively (Table 6). The analysis of the binding conformations revealed that Tetradioxin infiltrates the binding sites of each key gene, with abundant hydrogen bond donors and acceptors surrounding the binding sites (Fig. 5d).

**Table 6:** Binding energy of molecular docking

gene	drug	binding energy(kcal/mol)
FLT3LG	Tetradioxin	-6.3
ITGB7	Tetradioxin	-7.0
PARP1	Tetradioxin	-6.3

### The manifestation of key genes in AMI and control specimens was investigated

**Fig. 6:**
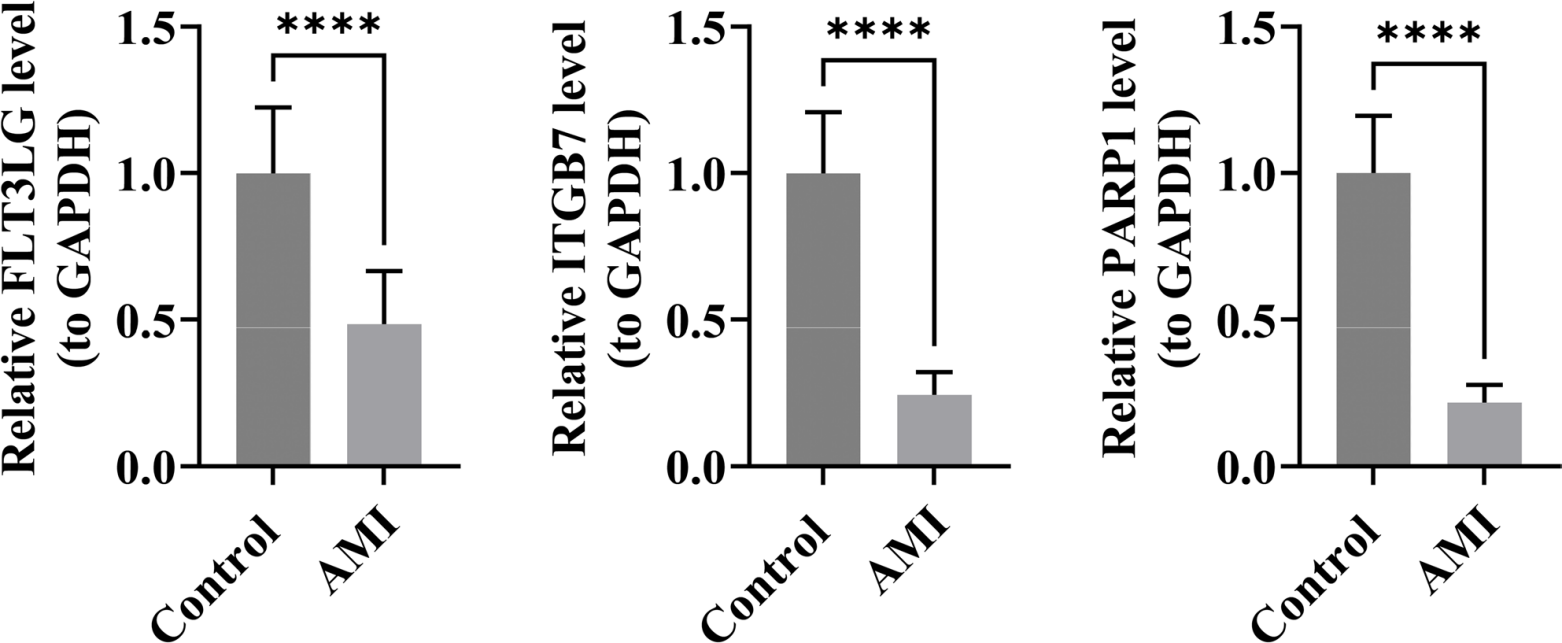
The expression levels of the three key genes were measured in clinical samples. FLT3LG, ITGB7, and PARP1 were significantly underexpressed in AMI samples **** (P-value < 0.0001)

The expression levels of the three key genes were measured in clinical samples. The results revealed that FLT3LG, ITGB7, and PARP1 were significantly underexpressed in AMI samples (P-value < 0.0001) (Fig. 6). These findings are consistent with the bioinformatics analysis, confirming the reliability of the bioinformatics results.

## Discussion

AMI is a severe cardiovascular condition that poses a significant threat to human health. It has been reported that differential metabolites in myocardial infarction are primarily products of the NAM metabolic pathway, suggesting that NAM metabolism may play a critical role in AMI pathogenesis [8]. However, this hypothesis requires further experimental validation. This study utilized GEO data on AMI, employing methods such as differential expression analysis and MR analysis to explore the role of key NMRGs in AMI. The findings provide new potential targets and a theoretical basis for diagnosing and treating AMI. In this study, FLT3LG, ITGB7, and PARP1 were identified as robust candidate NMRGs in AMI. It should be noted that all the analyses in this study were based on peripheral blood samples, and the results mainly reflect the systemic response of the body to AMI rather than the pathological processes within the myocardial tissue. Therefore, the specific mechanisms of action of these genes in myocardial injury still need to be further explored and verified in combination with myocardial tissue or animal models.

FLT3LG, or FMS-related receptor tyrosine kinase 3 ligand, is closely associated with DCs and T cells. FLT3LG promotes DC differentiation, expansion, and antigen-presenting capacity by binding to the FLT3 receptor on the surface of DCs [37]. It also activates T cells through the FLT3-FLT3LG signaling axis [38, 39]. AMI is strongly associated with both DCs and T cells. In AMI, circulating DC precursors (DCPs) are significantly reduced, with the reduction being more pronounced in patients with ST-segment elevation myocardial infarction (STEMI) compared to those with non-ST-segment elevation myocardial infarction (NSTEMI). Concurrently, inflammatory serum cytokines in patients with AMI are elevated, likely due to the recruitment of DCPs to the infarcted myocardium [40]. In a mouse model of AMI, DC infiltration was observed in the infarcted myocardium [41]. Additionally, one study demonstrated that CCR7 mediates the migration of DC-derived exosomes, improving cardiac function post-myocardial infarction [41]. After AMI, CD4^+^ T cells are activated, with cytokines such as IL-4 and IL-6 secreted by these cells playing a role in inflammatory regulation [42]. CD4^+^ T cell-derived exosomes can exacerbate myocardial fibrosis [42], while CD8^+^ T cells infiltrate the infarcted myocardium and worsen inflammation [43]. The balance between CD8^+^ T cells and regulatory T cells is critical for immune homeostasis [44]. Therefore, FLT3LG may contribute to the onset and progression of myocardial infarction by regulating the immune functions of DCs and T cells.

The ITGB7 gene plays a multifaceted role in various diseases, exhibiting both biological functions and clinical significance. In breast cancer, ITGB7 contributes to tumor progression by facilitating cell-matrix adhesion and membrane localization. Its expression is significantly elevated in tumor tissues and is linked to the menopausal status of patients. Through its integrin-binding function, ITGB7 mediates essential molecular pathways, including focal adhesion, ECM-receptor interaction, and the PI3K-Akt signaling pathway, potentially serving as a prognostic marker for breast cancer [45]. In skin inflammatory diseases such as psoriasis and lichen planus, serum levels of ITGB7 do not significantly correlate with disease activity or liver pathology. However, the abnormal expression of its ligand MAdCAM-1 suggests that this pathway may indirectly participate in inflammatory regulation [46]. In atherosclerosis, ITGB7 has been identified as a core gene related to immune infiltration in carotid plaques, implicating its potential role in regulating the inflammatory microenvironment. The precise mechanism, however, remains to be fully explored [47]. These studies suggest that ITGB7 has pleiotropic effects in both tumor biology and immune-related diseases, with its mode of action varying depending on the disease type. Its regulation of the cancer microenvironment and immune cell interactions warrants further investigation.

PARP1 (Poly(ADP-ribose) Polymerase 1) has a dual role in cardiac pathophysiology, with context-dependent effects on myocardial repair and injury. After myocardial infarction, PARP1 promotes cardiomyocyte proliferation and heart regeneration by interacting with HSP90AB1, enhancing its binding to CDC37, and activating cell cycle kinases, thus aiding in cardiac functional recovery [48]. However, PARP1 overactivation can contribute to adverse outcomes in ischemic cardiac conditions. For example, complement factor D (CFD) from epicardial adipose tissue exacerbates cardiomyocyte apoptosis after infarction through PARP1 hyperactivation, a process that can be mitigated by CFD or PARP1 inhibition [49]. Additionally, during myocardial ischemia-reperfusion injury, PARP1 activation drives autophagy-mediated cardiomyocyte death. Suppressing PARP1 *via* Sp1 transcriptional regulation or pharmacological inhibition reduces this damage [50]. These findings highlight PARP1’s dual regulatory function—enhancing regenerative potential under controlled conditions while exacerbating injury through apoptosis and autophagy under pathological stress. This dual nature positions PARP1 as a key therapeutic target that requires context-specific modulation in cardiac diseases.

MR analysis revealed a significant causal relationship between three key genes- FLT3LG, ITGB7, and PARP1-and AMI. These genes may influence the occurrence and development of AMI through modulation of nicotinamide metabolism, OXPHOS, immune responses as well as PI3K-Akt signaling pathways. Notably, genetic evidence identifies FLT3LG and ITGB7 as risk factors for AMI, while PARP1 acts as a protective factor. Paradoxically, all three genes were found to be downregulated in blood samples from AMI patients. This apparent contradiction may point to the fundamentally different biological interpretations of genetically determined baseline expression versus acute-phase dynamic fluctuations. Genetic effects operate over a lifetime to set the basal tone, while acute pathological conditions trigger rapid, often compensatory transcriptional responses that may mask or even reverse the underlying genetic predisposition. Specifically, PARP1 emerged as a protective factor, potentially through its physiological role in mitigating oxidative stress-induced DNA damage via efficient repair mechanisms, while simultaneously maintaining NAD⁺ pools to preserve mitochondrial oxidative phosphorylation and cellular energetics [51, 52]. However, during the acute phase of AMI, intense oxidative stress can lead to hyperactivation of PARP1. In individuals with genetically determined lower baseline expression levels, their repair capacity may be insufficient to cope with this overactivation, paradoxically exacerbating myocardial injury through NAD⁺ depletion and subsequent energy crisis [53]. Conversely, FLT3LG and ITGB7 were identified as risk factors, suggesting that their higher genetically determined expression levels may predispose individuals to a more active immune state or a chronic low-grade inflammatory environment. Long-term and adverse environmental factors synergistically increase the cumulative risk of AMI [54]. During the acute phase of AMI, however, local ischemia and the ensuing inflammatory storm can directly suppress FLT3LG synthesis. Concurrently, the massive recruitment of its consumers—specifically, dendritic cell precursors—to the infarcted region leads to a measurable decrease in its peripheral blood levels [citation:39]. Concurrently, acute oxidative stress may downregulate ITGB7 through epigenetic mechanisms, thereby impairing cardiomyocyte survival and repair capacity mediated by the PI3K-Akt signaling pathway [55, 56]. In summary, maintaining homeostasis and resilience of key cellular pathways is crucial for the prevention of AMI. However, MR analysis reveals the causal effect of lifelong genetically determined gene expression levels on the cumulative risk of AMI, rather than the dynamic roles of these genes during acute events. Future studies should employ longitudinal designs to dynamically monitor changes in gene expression before, during, and after AMI. Additionally, the specific regulatory mechanisms of these genes in nicotinamide metabolism and cardiac repair should be further elucidated using myocardial in situ models. These integrated approaches will help achieve a more comprehensive understanding of the intrinsic association between genetic factors and AMI pathogenesis.

According to GSEA, three key genes were significantly enriched in several pathways, including olfactory transduction, ribosome biogenesis, oxidative phosphorylation (OXPHOS), Huntington’s disease, and the spliceosome. In contrast, the direct involvement of pathways like olfactory transduction and Huntington’s disease in AMI pathogenesis remains ambiguous, potentially reflecting noise or false positives. However, the robust enrichment of the OXPHOS pathway is particularly compelling, underscoring its pivotal role in cardiac energy metabolism. During AMI, the efficiency of myocardial mitochondrial OXPHOS is significantly impaired. Clinical studies have shown that patients with AMI exhibit a marked reduction in leukocyte mitochondrial oxygen consumption and OXPHOS coupling compared to healthy individuals [57], suggesting that impaired respiratory chain function may exacerbate energy metabolism disorders. Acute ischemia triggers sympathetic overactivation and norepinephrine release, which further suppress mitochondrial OXPHOS through β-adrenergic receptor signaling. Notably, the β-blocker propranolol can prevent mitochondrial K^+^ efflux and restore OXPHOS coupling [58], highlighting sympathetic activation as a key driver of OXPHOS dysfunction. During ischemia-reperfusion, abnormal OXPHOS leads to excessive ROS production [59]. Notably, NAM metabolism is functionally coupled with OXPHOS, primarily through NAD^+^, the core coenzyme of OXPHOS that regulates energy metabolism [60, 61]. NAM, as a key substrate in the NAD^+^ salvage pathway, can be converted into NAD^+^ by enzymes such as NAM phosphoribosyltransferase (NAMPT). NAD^+^, a critical coenzyme of OXPHOS, is essential for maintaining OXPHOS efficiency and regulating energy metabolism [62]. Cellular NAD^+^ deficiency, often caused by oxidative stress or pathological conditions, significantly reduces OXPHOS efficiency, leading to an energy crisis [63, 64]. NAM replenishes NAD^+^ pools through salvage pathways to sustain OXPHOS function. For instance, in glaucoma models, NAM enhances retinal ganglion cell OXPHOS activity and improves mitochondrial function to counteract metabolic stress [65]. These findings highlight the vital role of OXPHOS in both AMI and ischemia-reperfusion injury and its intricate relationship with NAM metabolism.

In exploring potential treatments for AMI, drug prediction results suggest that Tetradioxin exhibits the strongest correlation with the three key genes (FLT3LG, ITGB7, and PARP1). Molecular docking studies indicate binding energies of -6.3, -7.0, and − 6.3 kcal/mol, respectively, for these genes with Tetradioxin’s active ingredients. Tetradioxin has shown potential therapeutic effects in various diseases. In COVID-19 individuals with asthma and vitamin D deficiency [66], Tetradioxin was shown to suppress pro-inflammatory cytokine production. For ulcerative colitis [67], Tetradioxin was predicted as a potential treatment drug, acting through SLC26A2 to exert anti-inflammatory effects. In the context of myocardial infarction, Tetradioxin may exert its effects through anti-inflammatory and cell cycle-regulating mechanisms.

However, it must be emphasized that these predicted results are currently based primarily on bioinformatics analysis and lack direct biological context and experimental support. Therefore, the potential role of Tetradioxin in AMI remains to be further verified, and its specific mechanisms and therapeutic value need to be elucidated through in-depth in vitro and in vivo studies.

This study identified three key genes (FLT3LG, ITGB7, and PARP1) associated with NAM metabolism in AMI through bioinformatics analysis. RT-qPCR validation confirmed their significant downregulation in the AMI group compared to controls, consistent with the bioinformatics results. Mechanistic analysis suggests that these genes may influence AMI pathogenesis through specific metabolic pathways and show potential interactions with immune cells. However, several limitations need to be considered. First, the public datasets and internal validation cohorts used in this study have limited sample sizes, which may affect the stability and generalizability of the models and findings. Second, predictive models (such as nomograms) constructed based on limited samples may be prone to result fluctuations and overfitting risks. Additionally, the current conclusions are primarily derived from bioinformatics analysis and genetic inference, lacking experimental-level functional validation. Finally, Significant heterogeneity was observed for certain genes (e.g., FLT3LG and STAT4) in the MR analysis, whilst the limited number of instrumental variables (*n* = 3) for STAT4 and ZNF395 may compromise the robustness of causal associations. It is particularly important to note that the study mainly relied on blood eQTL data and clinical samples, which may not fully represent gene expression and pathophysiological states in cardiac tissues—especially in cardiomyocytes and the local immune microenvironment. In the future, we plan to validate the core findings of this study in larger-scale, phenotypically homogeneous independent cohorts and utilize gain- and loss-of-function experiments in cellular and animal models to clarify the roles of key genes in myocardial injury and immune regulation. Furthermore, we aim to integrate multi-omics technologies—such as single-cell transcriptomics and epigenomics—to analyze the expression dynamics and regulatory networks of these genes at a cell-type-specific level. This will systematically elucidate their specific mechanistic roles in the occurrence and progression of AMI.

## Conclusion

This study identified FLT3LG, ITGB7, and PARP1 as key NMRGs in AMI, with FLT3LG and ITGB7 serving as risk factors and PARP1 as a protective factor. These genes were consistently downregulated in patients with AMI and linked to critical pathways such as OXPHOS and immune regulation, particularly neutrophil infiltration. Tetradioxin emerged as a potential therapeutic candidate due to its strong binding affinity with these genes. While further validation is needed, these findings offer novel insights into the mechanisms of AMI and highlight promising diagnostic and therapeutic targets for improving AMI management.

## Supplementary Information

Supplementary Material 1. List of 42 NMRGs.

Supplementary Material 2. List of 297 key module genes related to nicotinamide metabolism in acute myocardial infarction identified through WGCNA.

Supplementary Material 3. Scatter plots of Mendelian randomization analysis for 17 exposure factors associated with AMI risk.

Supplementary Material 4. Forest plots of Mendelian randomization effect estimates for 17 exposure factors.

Supplementary Material 5. Funnel plots assessing instrumental variable validity in Mendelian randomization analysis.

Supplementary Material 6. Leave-one-out sensitivity analysis of Mendelian randomization results.

Supplementary Material 7. GO enrichment analysis results of feature genes.

Supplementary Material 8. KEGG pathway enrichment analysis results of feature genes in AMI.

Supplementary Material 9. GSEA results of FLT3LG in AMI.

Supplementary Material 10. GSEA results of ITGB7 in AMI.

Supplementary Material 11. GSEA results of PARP1 in AMI.

Supplementary Material 12. GSVA enrichment scores of key genes across metabolic pathways in AMI and control samples from the training set.

## Data Availability

The datasets analysed during the current study are available in the Gene Expression Omnibus (GEO) database repository, [http://www.ncbi.nlm.nih.gov/geo/], and the Integrative Epidemiology Unit Open GWAS (IEU OpenGWAS) database repository, [https://gwas.mrcieu.ac.uk/].

## Abbreviations

Abbreviations: Full Names
AMI: Acute myocardial infarction
MR: Mendelian randomization
GSEA: Gene set enrichment analysis
ssGSEA: single-sample Gene Set Enrichment Analysis
OR: Odds ratio
cor: Correlation coefficient
ECGs: Electrocardiograms
NAM: Nicotinamide
NAD: Nicotinamide adenine dinucleotide
NADP: Nicotinamide adenine dinucleotide phosphate
SNPs: Single nucleotide polymorphisms
GEO: Gene expression omnibus
NMRGs: Nicotinamide metabolism-related genes
GWAS: Genome-wide association study
IEU OpenGWAS: Integrative epidemiology unit open GWAS
eQTLs: Expression quantitative trait loci
DEGs: Differentially expressed genes
MM: Module membership
KEGG: Kyoto encyclopedia of genes and genomes
GO: Gene ontology
DMNC: Density of maximum neighborhood component
MCC: Maximum clique centrality
EPC: Edge percolated component
NCBI: National center for biotechnology information
DCA: Decision curve analysis
MSigDB: Molecular signatures database
NES: Normalized enrichment score
DSigDB: Drug signatures database
RCSB PDB: RCSB protein data bank
RT-qPCR: Reverse transcription quantitative polymerase chain reaction
DCs: Dendritic cells
DCPs: Dendritic cell precursors
STEMI: ST-segment elevation myocardial infarction
NSTEMI: Non-ST-segment elevation myocardial infarction
CFD: Complement factor D
OXPHOS: Oxidative phosphorylation
NAD+: Nicotinamide adenine dinucleotide
NAMPT: Nicotinamide phosphoribosyltransferase
