# Supplementary figures and images for: Recognition and confirmation of key genes associated with nicotinamide metabolism in acute myocardial infarction
Source: Hereditas. 2026 Mar 29;163:58. doi: 10.1186/s41065-026-00671-0 (PMC13154459; doi:10.1186/s41065-026-00671-0)

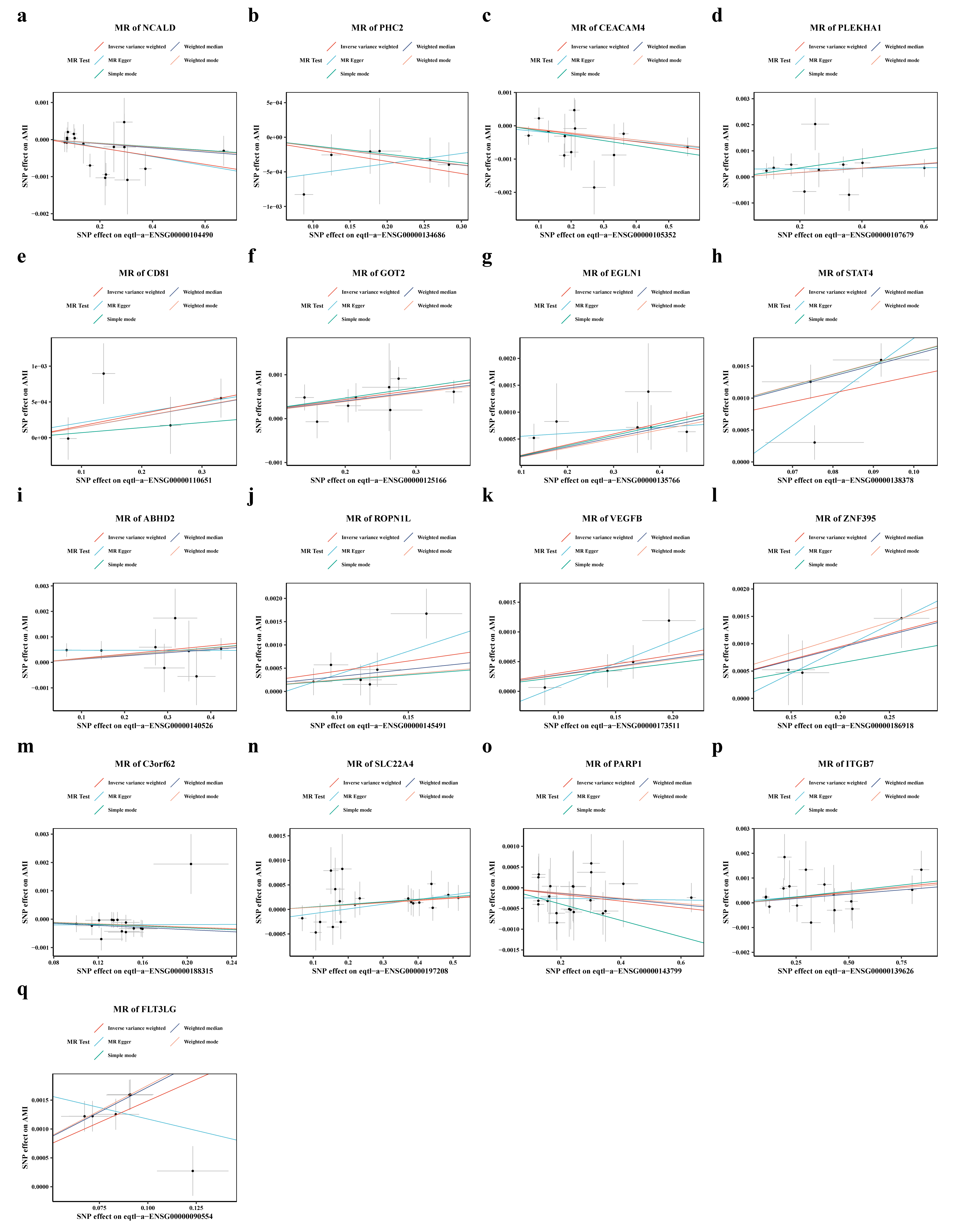

Supplement: Supplementary file 3 — Supplementary Material 3. Scatter plots of Mendelian randomization analysis for 17 exposure factors associated with AMI risk. [file 41065_2026_671_MOESM3_ESM.tif]

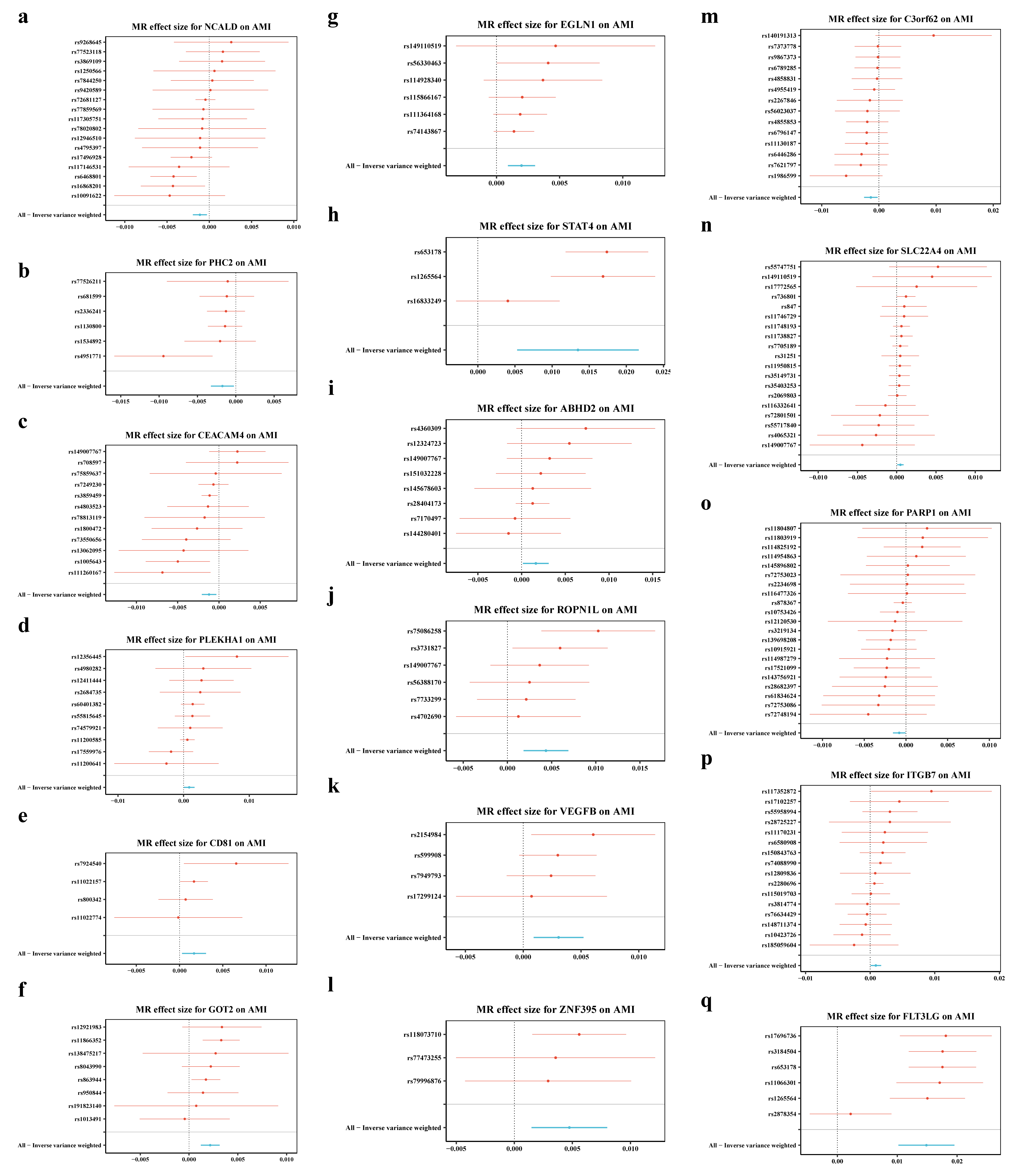

Supplement: Supplementary file 4 — Supplementary Material 4. Forest plots of Mendelian randomization effect estimates for 17 exposure factors. [file 41065_2026_671_MOESM4_ESM.tif]

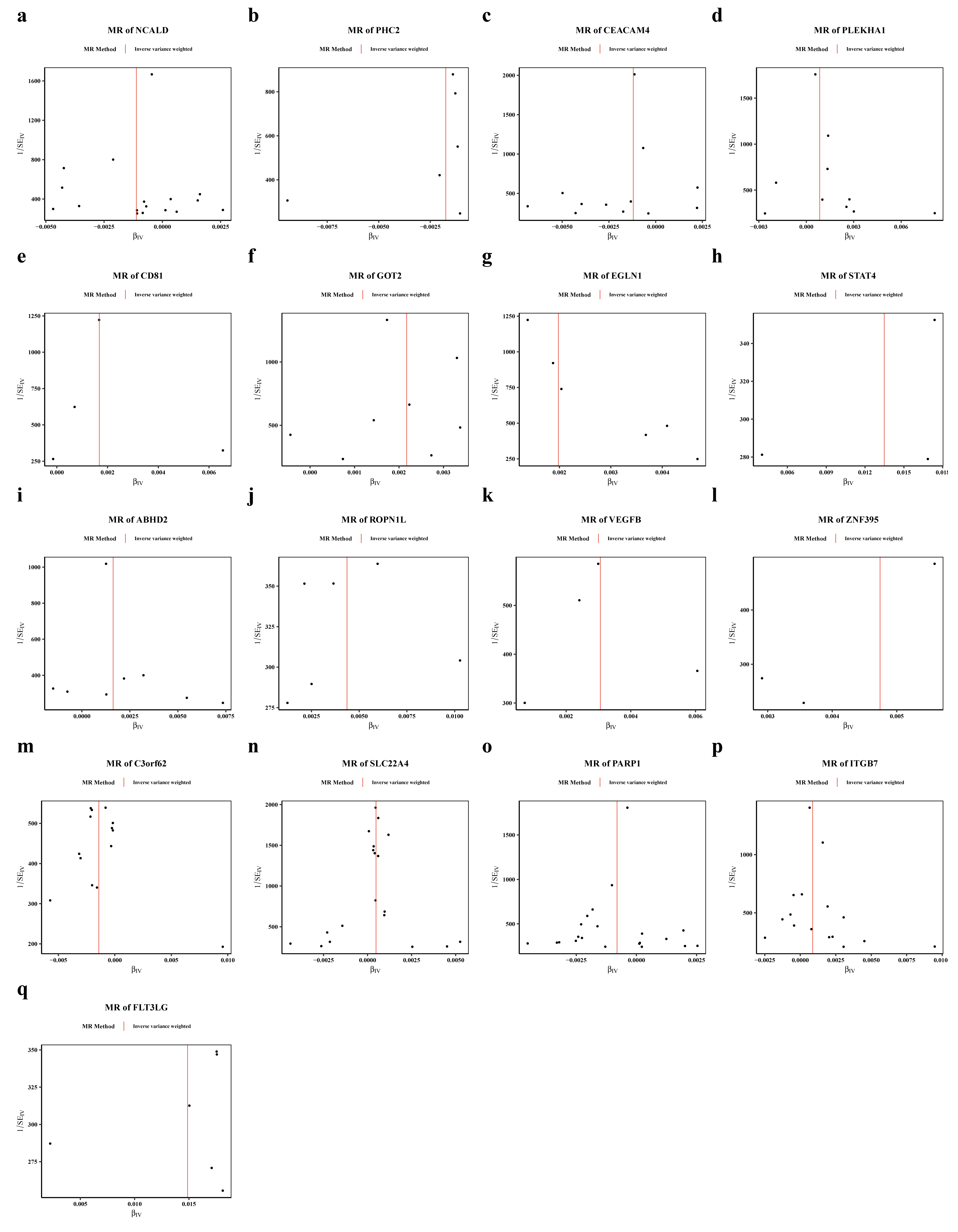

Supplement: Supplementary file 5 — Supplementary Material 5. Funnel plots assessing instrumental variable validity in Mendelian randomization analysis. [file 41065_2026_671_MOESM5_ESM.tif]

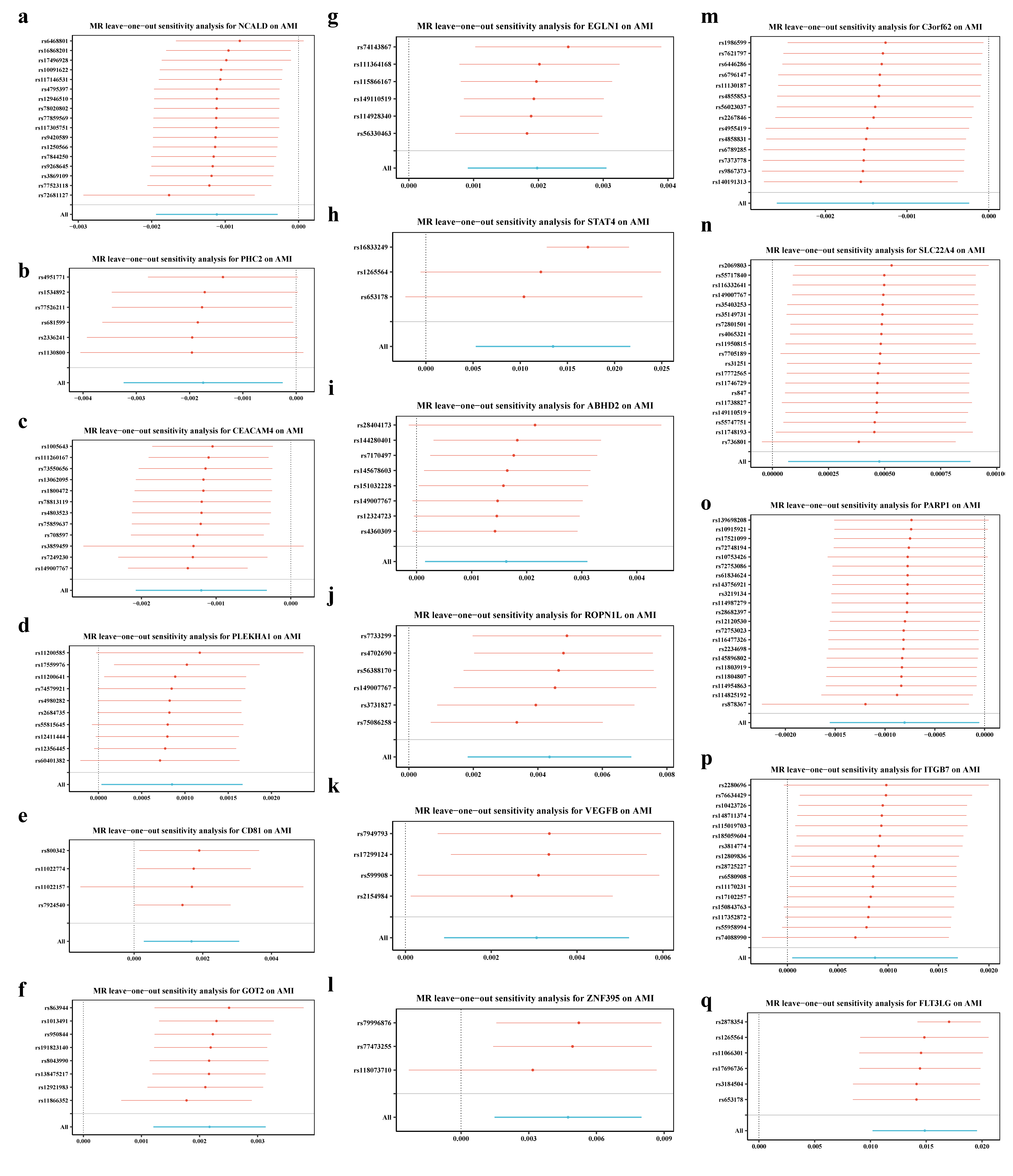

Supplement: Supplementary file 6 — Supplementary Material 6. Leave-one-out sensitivity analysis of Mendelian randomization results. [file 41065_2026_671_MOESM6_ESM.tif]
